# Editorial: Bioinformatics Analysis of Single Cell Sequencing Data and Applications in Precision Medicine

**DOI:** 10.3389/fgene.2019.01358

**Published:** 2020-01-23

**Authors:** Jialiang Yang, Bo Liao, Tuo Zhang, Yifei Xu

**Affiliations:** ^1^ School of Mathematics and Statistics, Hainan Normal University, Haikou, China; ^2^ Department of Sciences, Geneis Beijing Co., Ltd., Beijing, China; ^3^ Department of Microbiology and Immunology, Weill Cornell Medicine, New York, NY, United States; ^4^ Nuffield Department of Medicine, University of Oxford, Oxford, United Kingdom

**Keywords:** single cell RNA sequencing, bioinformatics, precision medicine, cell cluster, trajectory analyses

Next-generation sequencing (NGS) technology has been successfully applied in disease diagnostics, oncological immunotherapy, and drug repurposing, especially for precision medicine where optimized medication is tailored to individual patients. Recently, the development of single cell techniques makes it possible to examine gene expression and mutation at individual cell resolution, which provides an unprecedented opportunity to study cell development and differentiation, and reveal cell-to-cell heterogeneity during disease development, treatment, and drug response for individual patients. With the exponential increase of single cell sequencing data, it is critical to develop appropriate bioinformatics and machine learning tools to mine the rules behind them. However, due to the technical barriers in single cell sequencing and the noisy nature of raw sequencing data, this task is challenging especially in the context of disease diagnosis and drug development.

To promote the translation and efficient usage of single cell sequencing data to precision medicine, it is necessary to develop new analysis tools for analyzing and integrating multi-level single cell data including DNA, RNA, protein, and so on, comparing existing methods and results derived from different studies, and enhancing disease diagnostics and drug development. For example, the quality control, normalization, differential gene calling, and clustering methods are quite different between single cell sequencing and traditional bulk cell sequencing. Thus, it is critical to develop a best practice specifically for dealing with single cell sequencing data. For disease treatment, it is also important to identify disease driver genes common to all cell types as well as those specific to a particular cell type or subgroup as revealed by single cell techniques, based on existing or novel network and machine learning-based methods. Finally, more translational work should be done to bridge the bioinformatics analyses and clinical applications for single cell researchers.

To provide a platform bridging single cell analysis and translational studies, we organized this special issue, in which 11 manuscripts have been accepted for publication. Firstly, Zen and Dai presented a comprehensive review on scRNA-seq associated biological experiments as well as computational methods for evaluating disease heterogeneity. They described the early impact of such technologies as well as a variety of common methods applicable to upstream and downstream processes. Upstream processes include several computational methods related to the detection and removal of technical noise given commonly assumed statistical distributions. In addition, the authors overviewed the recent adoption of methods to combat the statistical effects of batched experiments and zero-inflated data. Downstream processes include methods to integrate transcriptomic information with several other types of data such as epigenetic or spatial. They also introduced several clustering and pseudotemporal ordering methods. Finally, the authors conducted a small study comparing a handful of clustering and pseudotemporal analysis methods on four marginally related datasets due to their significance to disease systems.

Z. Wang et al. introduced a newly built database, SCDevDB, which provides the analysis results of single-cell gene expression profiles in different human developmental processes. This database mainly contains the gene expression profiles across 35 development stages as well as the differential gene analysis for 24 developmental pathways.

The manuscript by Finnegan et al. utilized single-cell RNA-seq data from 22,338 human foreskin keratinocytes to study transcription factor networks during the keratinocyte transition from the basal to the differentiated state. Their analysis uncovered novel players and novel roles of transcription factors in the intricate orchestration of keratinocyte differentiation and shed lights in elucidating disease and cancer processes.

Wu et al. designed a framework for evaluating 14 commonly used gene expression normalization methods, achieving consistency in the evaluation results using both bulk RNA-seq and scRNA-seq data. This framework was implemented as R package for researchers to choose the best normalization method.

X. Wang et al. identified 91 ethylene-responsive factors (ERFs) in *F. vesca*, based on which they provided evolutionary analysis, expansion analysis and expression analysis, especially for the influences of tandem duplication mechanism on expansion of ERF gene family.

Yin et al. focused on identification of novel breast cancer predisposition genes, which is of great significance in understanding the pathogenesis of breast cancer. The authors reanalyzed published whole exon sequencing data to screen susceptible genes, followed with experimental and functional validation. The most striking finding in the article is the discovery of *NCK1* as a novel breast cancer gene and the authors successfully correlated its expression and function with carcinogenesis.

Bope et al. provides a comprehensive review of genomic resources that have been established with respect to African individuals and their genomic data. This review presents an interesting perspective, and road map concerning the developments and studies that are needed in order to complement and promote efforts related to implementing Clinical Genomics in Africa.

Mercatelli et al. conducted a pan-cancer analysis to investigate the predictive power of gene expression on somatic mutations and copy number variations. They showed that genomic alterations could be modeled by gene expression across several human cancers using machine learning algorithms, and single-cell sequencing data can increase the performance of the model.

Chen et al. investigated the gene expression profiles of patient-derived tumor xenograft (PDX) models originated from eight tissues using machine learning algorithms, and showed that the specificity of primary tumor site was preserved in PDX models.

Cheng et al. provided a comprehensive review of recently technologies and literature of human microbiome. Firstly, the technologies producing the microbiome big data were reviewed. Secondly, the connections of the microbiota with different host organs were discussed. After that, the association of microbiota with the clinical medicine was discussed, with a special focus on a few major microbiota-associated diseases. Lastly, the future research trends were proposed.

Liu et al. conducted an integrated bioinformatics analysis on the public epithelial ovarian cancer (EOC) data collected from GEO; they identified potential biomarkers for evaluating EOC prognosis and bioactivate compounds for EOC treatment. Their study provides an example of bioinformatics analysis in promoting cancer research.

## Author Contributions

JY, BL, TZ and YX organized this special issue and wrote the editorial. All authors have approved the final version of the editorial.

## Conflict of Interest

JY is currently employed by Geneis Beijing Co. Ltd.

The remaining authors declare that the research was conducted in the absence of any commercial or financial relationships that could be construed as a potential conflict of interest.

